# Supplementary material for: Randomization‐Based Inference for MCP‐Mod
Source: Stat Med. 2025 May 22;44(10-12):e70092. doi: 10.1002/sim.70092 (PMC12097294; doi:10.1002/sim.70092)
Supplement: Supplementary file 1 — CODE. Code is publicly available [43]. [file SIM-44-0-s002.pdf]

SUPPLEMENTARY MATERIAL: CODE

MCP_Rand.R

```
library(DoseFinding)
library(ggplot2)
library(logistf)
library(dplyr)
library(broom)

### logit Functions ###################################
logit <- function(p) log(p / (1 - p))
inv_logit <- function(y) 1 / (1 + exp(-y))

#### Permuted Block Randomization ##################################
# This method is rerandomizing a sequence that was created PBR
### x is the full randomization sequence that we which to randomize
### B is the block size
PBR <- function(x, B=7){
  num_blocks <- round(length(x)/B)
  full_blocks <- num_blocks == round(length(x)/B)
  for(i in 1:num_blocks){
    x[((i-1)*B+1):(i*B)] <- sample(x[((i-1)*B+1):(i*B)])
  }
  if(!full_blocks){
    x[num_blocks*B+1:length(x)] <- sample(x[num_blocks*B+1:length(x)])
  }
  return(x)
}

#################### Generating Data Example Function ##########################
generate_data <-function(seed=20, doses, mods, n, randMethod="PBR", B=7, x1,
                          timetrend=FALSE, p1=0.2, pK=0.2){
  #### Data Generation ##################################
  set.seed(seed, kind = "Mersenne-Twister", sample.kind = "Rejection",
  normal.kind = "Inversion")
  dose_block <- c(0,10,10,25,25,100,100)
  if(randMethod=="CR"){
    dose_vector <- sample(doses, size =sum(n), replace = T, prob=n/sum(n))
  } else if (randMethod=="RA"){
    dose_vector <- rep(dose_block,sum(n)/B)
    dose_vector <- sample(dose_vector)
  } else if (randMethod=="PBR") {
    dose_vector <- rep(dose_block,sum(n)/B)
    dose_vector <- PBR(dose_vector, B)
  }
  ## emax cal
  emax_max=(logit(pK)-logit(p1))*110/100
  ## Covariate
  prob <- inv_logit(emax(dose_vector, logit(p1), emax_max, 10) + 0.6*x1 )
  ##### Time Trend ####
  if(timetrend){
```

```
    prob <- prob + seq(-0.2,0.2,length.out=sum(n))
  }
  prob[prob > 1] <- 1; prob[prob < 0] <- 0   # Move time trend to logit-scale
  dat <- data.frame(y = rbinom(sum(n), 1, prob), dose = dose_vector)
  return(dat)
}


#reg_mod return mean and covariance matrix or residuals
#allows for glm and logistf
#pearson & working residuals are commented out but can be used for future research
reg_mod <- function(reg="glm", covariates = FALSE, test_type="population", dat, x1,
                    doses){
  if(test_type=="randomisation_residuals"){
    if(reg=="glm" & !covariates){
      fit <- glm(y~1 , data = dat, family = binomial)
      ### Pearson Residuals
      #residus <- residuals(fit, type="pearson")
      ### Response Residuals
      residus <- residuals(fit, type="response")
      ### Working Residuals
      #residus <- residuals(fit, type="working")
    } else if(reg=="logistf" & !covariates){
      fit <- logistf(y ~ 1, data = dat)
      ### Pearson Residuals
      #res <- dat$y - fit$predict
      #residus <- res/(sqrt(fit$hat.diag))
      ### Response Residuals
      residus <- dat$y - fit$predict
      ### Working Residuals
      #res <- dat$y - fit$predict
      #residus <- res/(fit$hat.diag)
    } else if(reg=="glm" & covariates){
      fit <- glm(y~1 + x1 , data = dat, family = binomial)
      ### Pearson Residuals
      #residus <- residuals(fit, type="pearson")
      ### Response Residuals
      residus <- residuals(fit, type="response")
      ### Working Residuals
      #residus <- residuals(fit, type="working")
    } else if(reg=="logistf" & covariates){
      fit <- logistf(y ~ 1+ x1, data = dat)
      ### Pearson Residuals
      #res <- dat$y - fit$predict
      #residus <- res/(sqrt(fit$hat.diag))
      ### Response Residuals
      residus <- dat$y - fit$predict
      ### Working Residuals
      #res <- dat$y - fit$predict
      #residus <- res/(fit$hat.diag)
    }
```

```
    return(list(residus=residus))
  } else {
    if(reg=="glm" & !covariates){
      fit <- glm(y ~ factor(dose) + 0, data = dat, family = binomial)
      mu_hat <- coef(fit); S_hat <- vcov(fit)
    } else if(reg=="logistf"  & !covariates){
      fit <- logistf(y ~ factor(dose) + 0, data = dat)
      mu_hat <- fit$coefficients; S_hat <- fit$var
    } else if(reg=="glm" & covariates){
      fit <- glm(y ~ factor(dose) + x1 -1, data = dat, family = binomial)
      mu_hat <- coef(fit)[1:length(doses)]
      S_hat <- vcov(fit)[1:length(doses),1:length(doses)]
    } else if(reg=="logistf"  & covariates){
      fit <- logistf(y ~ factor(dose) + x1 - 1, data = dat)
      mu_hat <- fit$coefficients[1:length(doses)]
      S_hat <- fit$var[1:length(doses),1:length(doses)]
    }
  }

  return(list(mu_hat=mu_hat, S_hat=S_hat))
}

######### MCP Test based on Response Residuals ##################################
MCP_RD <- function(dat, doses, models, contMat, residus, n) {
  r_mean <- tapply(residus, dat$dose, mean)
  sZ_mean <- tapply(residus, dat$dose, var)

  SZ <- apply(contMat, 2, function(col) {
    sum(col * r_mean) / sqrt(sum(col^2 * sZ_mean / n))
  })

  return(SZ)
}


### rerandomizing randomisation sequence
get_randomised_data <- function(dat, randMethod, doses, B, N, n) {
  dose_vector <- switch(randMethod,
                        "CR" = sample(doses, size = N, replace = TRUE, prob = n/N),
                        "RA" = sample(dat$dose),
                        "PBR" = PBR(dat$dose, B))
  dat$dose <- dose_vector
  return(dat)
}

MCP_Rand <- function(dat=dat, doses=doses,  n=n, test_type="population",
                     reg="glm", randMethod="PBR", B=7, x1, covariate=FALSE,
                     nrand=1000){
  doses <- c(0, 10, 25, 100)
  mods <- DoseFinding::Mods(emax = c(10, 50),
                            sigEmax = rbind(c(5, 3), c(25, 3)),
```

```
                                   #betaMod = c(0.2475, 2.025),    # orginal betaMod
                                   betaMod = c(0.1529, 0.5809),    # new betaMod
                                   doses = doses)
  reg_val <- reg_mod(dat=dat, reg=reg, x1=x1, test_type=test_type,
                               covariates=covariate, doses)
  if(test_type=="population"){
    pvals <- min(attr(MCTtest(dose=doses, resp=reg_val$mu_hat, S=reg_val$S_hat,
                               models = mods, alternative = "one.sided", alpha =0.1,
                               type = "general")$tStat,"pVal"))
  } else if(test_type=="randomisation_mean"){
    results <- rep(0, nrand + 1)
    results[1] <- max(MCTtest(doses, reg_val$mu_hat, S = reg_val$S_hat,
                  models = mods, type = "general", pVal = FALSE, critV = 0)$tStat)
    for(j in 1:nrand){
      datR <- get_randomised_data(dat[, 1:2], randMethod, doses, B, sum(n), n)
      rand_reg_val <- reg_mod(dat=datR, reg=reg, x1=x1, test_type=test_type,
                  covariates=covariate, doses)
      results[j+1] <- max(MCTtest(doses, rand_reg_val$mu_hat,
                  S = rand_reg_val$S_hat, models = mods, type = "general",
                  pVal = FALSE, critV = 0)$tStat)
    }
    pvals <- sum(results[-1]- results[1]>= 0 )/nrand
  } else if(test_type=="randomisation_residuals") {
    reg_val <- reg_mod(dat=dat, reg=reg, x1=x1, covariates=covariate,
                  doses, test_type="population")
    contMat <- optContr(models = mods, w=n)$contMat
    reg_val <- reg_mod(dat=dat, reg=reg, x1=x1, covariates=covariate,
                  test_type=test_type)
    residus <- reg_val$residus
    results <- rep(0, nrand + 1)
    results[1] <- max(MCP_RD(dat, doses, models, contMat, residus, n))
    for(j in 1:nrand){
      datR <- get_randomised_data(dat[, 1:2], randMethod, doses, B, sum(n), n)
      results[j+1] <- max(MCP_RD(datR, doses, models, contMat, residus, n))
    }
    pvals <- sum(results[-1]- results[1]>= 0 )/nrand
  }
  return(pvals)
}
```

runsimMP.R

```
    rm(list = ls())
usethis::edit_r_profile()
library(detectseparation)
library(clustermq)
library(DoseFinding)
library(ggplot2)
library(logistf)
library(dplyr)
```

```
library(broom)
library(tidyr)

## Set LSF as scheduler and path to template
options(clustermq.scheduler = "lsf")

source("MCP_Rand.R") ## load auxiliary code
export <- list(logit=logit, inv_logit=inv_logit, PBR = PBR,
               generate_data=generate_data, reg_mod=reg_mod, MCP_RD = MCP_RD,
               get_randomised_data=get_randomised_data, MCP_Rand=MCP_Rand,
               MCTtest=MCTtest, detect_separation=detect_separation)

## setup scenarios (toy example)
doses <- c(0, 10, 25, 100)
covariate <- c(TRUE)
reg <- c("glm", "logistf")
test_type <- c("population", "randomisation_mean", "randomisation_residuals")

# Create the full factorial grid first
params_changed <- expand.grid(test_type=test_type, reg=reg, covariate=covariate)

# Filter for MCP benchmark configurations only
filtered_params <- params_changed[!(params_changed$test_type == "population"  &
params_changed$reg !=  "glm"),]
filtered_params <- filtered_params[!(filtered_params$test_type == "population"  &
filtered_params$covariate ==  FALSE),]

# Convert remaining combinations to a data frame
filtered_params <- as.data.frame(filtered_params)

# Define the custom function
proportion_less_than_005 <- function(x) {
  # Remove NAs from the input vector
  x <- na.omit(x)
  if (length(x) == 0) return(NA) # Handle case where no data points left after
  # omitting NAs
  sum(x < 0.05) / length(x)
}

# Define the custom function
proportion_less_than_01 <- function(x) {
  # Remove NAs from the input vector
  x <- na.omit(x)
  if (length(x) == 0) return(NA) # Handle case where no data points left
  # after omitting NAs
  sum(x < 0.1) / length(x)
}

MCR_Rand_dat <- function(seed=1, randMethod= "PBR", doses=doses, n=n,  B=7, p1=0.2,
                pK=0.2, nrand=nrand, timetrend = FALSE,
                filtered_params=filtered_params){
```

```
  n_methods <- dim(filtered_params)[1]
  p_vals <- rep(0, n_methods)
  # Initialize run_time to store the runtime of each method
  run_time <- rep(0, n_methods)
  no_respon = FALSE; fail_MCP = FALSE; completesep = FALSE
  mods <- DoseFinding::Mods(emax = c(10, 50),
                            sigEmax = rbind(c(5, 3), c(25, 3)),
                            #betaMod = c(0.2475, 2.025),
                            betaMod = c(0.1529, 0.5809),
                            doses = doses)
  plotMods(mods, trafo = inv_logit)
  x1 <- rnorm(sum(n), 0, 1)
  dat <- generate_data(seed=seed, doses=doses, mods=mods, randMethod=randMethod,
            B=B, n=n, x1=x1, p1=p1, pK=pK, timetrend = timetrend )
  result <- glm(y ~ 1, data = dat, family = binomial, method = "detect_separation")
  completesep <- result$outcome

  if(sum(dat$y)==0){
   p_vals = rep(NA, n_methods)
   run_time = p_vals
  } else {
    for(i in 1:n_methods){
      if(sum(dat[dat$dose == 0,]$y)==0){completesep=TRUE}
      start_time <- Sys.time()  # Capture start time

      p_vals[i] <- MCP_Rand(dat=dat, doses=doses,  n=n,
                            test_type=filtered_params$test_type[i],
                            reg=filtered_params$reg[i],
                            randMethod="PBR", B=B, x1=x1,
                            covariate=filtered_params$covariate[i], nrand=nrand)

      end_time <- Sys.time()  # Capture end time

      run_time[i] <- end_time - start_time  # Calculate runtime
      if(filtered_params$test_type[i]=="population" & is.na(p_vals[i])){
        fail_MCP = TRUE
      }
    }
  }

  list(p_vals = p_vals, run_time = run_time, no_respon = no_respon,
       fail_MCP = fail_MCP, completesep = completesep)
       # Return both p-values and runtimes
}

simPlots <- function(results, paramscom, sim_matrix_p_vals=0){
  # Convert covariate to factor for better visualization
  results$reg <- factor(results$reg, labels = c("glm", "logistf"))

  # Add an additional column to combine "test_type" and "reg"
  # for purely visual purposes
```

```
results$combined <- interaction(results$test_type, results$reg)
# Defining the colors
colors <- c(rgb(215,25,28, maxColorValue = 255),
            rgb(253, 174, 97, maxColorValue = 255),
            rgb(255, 255, 51, maxColorValue = 255),
            rgb(171, 221, 164, maxColorValue = 255),
            rgb(43, 131, 186, maxColorValue = 255))
# Define the legend labels
methods <- c(
  "Population-based Test",
  "Randomisation Test (MLE)",
  "Randomisation Test (MLE; residual-based)",
  "Randomisation Test (penalized MLE)",
  "Randomisation Test (pen. MLE; residual-based)"
)
legend_labels <- methods


# Create the bar plot using ggplot
t1 <- ggplot(results, aes(x = combined, y = TypeIerror_Power*100,
              fill = combined)) +
  geom_bar(stat = "identity", position = "dodge", color = "black", size = 0.5) +
  scale_fill_manual(values = colors, labels = legend_labels) +
  labs(title = "Type I Error Rate for Different Tests",
       x = "", y = "Type I Error Rate in %",
       fill = "Legend") +
  ylim(0,12) +
  # Applying a minimal theme with larger base font size
  theme_minimal(base_size = 15) +
  theme(
    axis.text.x = element_blank(),           # Remove the text under each bar
    axis.ticks.x = element_blank(),          # Remove the ticks under each bar
    legend.title = element_blank(),          # Remove legend title
    legend.text.align = 0,                   # Align legend text
    # Center the title, increase size, and make it bold
    plot.title = element_text(hjust = 0.5, size = 18, face = "bold"),
    legend.position = "bottom",               # Position the legend to the right
    # Increase space between legend elements
    legend.spacing.y = unit(0.5, 'cm'),
    legend.text = element_text(size = 12)# Increase the legend text size
  ) +
  geom_text(aes(label = round(TypeIerror_Power*100, 3)),
            position = position_dodge(width = 0.9), vjust = -0.5, size = 4)
# Save the plot to a PDF file
ggsave(paste0("t1_", paramscom, ".pdf"), plot = t1, width = 14, height = 7)


# Create the bar plot using ggplot
ptt <- ggplot(results, aes(x = combined, y = RunTime, fill = combined)) +
  geom_bar(stat = "identity", position = "dodge") +
  scale_fill_manual(values = colors, labels = legend_labels) +
```

```
  labs(title = "RunTime for Different Tests",
       x = "",
       y = "RunTime (Seconds)",
       fill = "Test Type") +
  # Applying a minimal theme with larger base font size
  theme_minimal(base_size = 15) +
  theme(
    axis.text.x = element_blank(),            # Remove the text under each bar
    axis.ticks.x = element_blank(),           # Remove the ticks under each bar
    legend.title = element_blank(),           # Remove legend title
    legend.text.align = 0,                    # Align legend text
    # Center the title, increase size, and make it bold
    plot.title = element_text(hjust = 0.5, size = 18, face = "bold"),
    legend.position = "bottom",                # Position the legend to the right
    # Increase space between legend elements
    legend.spacing.y = unit(0.5, 'cm'),
    legend.text = element_text(size = 12)     # Increase the legend text size
  ) +
  geom_text(aes(label = round(RunTime, 3)),
            position = position_dodge(width = 0.9), vjust = -0.5, size = 4)

# Save the plot to a PDF file
ggsave(paste0("ptt_", paramscom, ".pdf"), plot = ptt, width = 11, height = 5)

# Convert matrix to long format
# df <- sim_matrix_p_vals[-1,]
df <- sim_matrix_p_vals
df <- df %>%
  # mutate(method = methods[-1]) %>%
  mutate(method = methods) %>%
  pivot_longer(cols = -method, names_to = "variable", values_to = "p_value")
# Factorize the method variable with specified levels' order
df <- df %>%
  mutate(method = factor(method, levels = methods))
# mutate(method = factor(method, levels = methods[-1]))

# Exclude NAs and create the plot
plot_pval <- ggplot(df %>% filter(!is.na(p_value)),
                  aes(x = p_value, fill = method)) +
  geom_histogram(bins = 50, alpha = 0.5, position = "identity", boundary = 0,
                  breaks = seq(0, 1, length.out = 51)) +
  scale_fill_manual(values = colors) +
  labs(title = "Histogram p-values by test", x = "P-value", y = "Frequency",
                  fill = "Test") +
  theme_minimal() +
  theme(legend.position = "bottom",
        plot.title = element_text(hjust = 0.5, size = 18, face = "bold")) +
  facet_wrap(~ method, scales = "free_y", ncol = 2)+ guides(fill="none")

ggsave(paste0("plot_pval_", paramscom, ".pdf"), plot = plot_pval,
                  width = 11, height = 8)
```

```
}

# Define the vectors
n <- c(7, 14, 14, 14)
time_trend <- c(TRUE, FALSE)
rand_method <- c("CR", "RA", "PBR")
sample_size <- list(n, 2*n, 10*n)
pK <- seq(0.2, 0.9, 0.1)

# Generate the combination grid
grid <- rbind(
      expand.grid(
      pK = pK,
      rand_method = rand_method[2:3],
      sample_size = I(sample_size[1]),
      time_trend = time_trend,
      KEEP.OUT.ATTRS = FALSE,
      stringsAsFactors = FALSE
    ),
    expand.grid(
      pK = c(0.61, seq(0.2, 0.7, 0.1)),
      rand_method = rand_method[2:3],
      sample_size = I(sample_size[2]),
      time_trend = time_trend,
      KEEP.OUT.ATTRS = FALSE,
      stringsAsFactors = FALSE
    ),
    expand.grid(
      pK = c(0.364,seq(0.2, 0.45, 0.05)),
      rand_method = rand_method,
      sample_size = I(sample_size[3]),
      time_trend = time_trend,
      KEEP.OUT.ATTRS = FALSE,
      stringsAsFactors = FALSE
    )
)

# Loop over all combinations
for (i in 1:nrow(grid)) {
  current_combination <- grid[i,]
  # Extracting current values (you can use these values in your tasks)
  current_time_trend <- current_combination$time_trend
  current_rand_method <- current_combination$rand_method
  current_sample_size <- current_combination$sample_size[[1]]
  current_pK <- current_combination$pK

  print(current_combination)

  # Create the full factorial grid first
  param_constatnt <- list(nrand=1000, doses=doses,
                    n=current_combination$sample_size[[1]],B=7,
```

```
                    timetrend = current_combination$time_trend,
                    pK= current_combination$pK,
                    randMethod= current_combination$rand_method,
                    filtered_params=filtered_params)

      # Number of Simulations
      nsim = 10000
      seeds <- data.frame(seed=seq(1,nsim))

      sim_out <- Q_rows(fun = MCR_Rand_dat,
                        df = seeds,
                        const = param_constatnt,
                        seed = 23456,
                        n_jobs = nrow(seeds),
                        template = list(
                          walltime = 300,
                          job_name = "MCP_Rand",
                          log_file = "logs.txt"),
                        pkgs = c("data.table", "DoseFinding", "logistf"),
                        export = export)

      paramscom <- paste("nsim", nsim, "nrand", param_constatnt$nrand, "pK",
                    round(current_combination$pK*100), "rand_method",
                    current_combination$rand_method, "N",
                    sum(current_combination$sample_size[[1]]),
                    'TimeTrend', current_time_trend, sep="_")
      filename <- paste("simulation", paramscom, "FULL", sep="_")
      saveRDS(sim_out, file= filename)

      percent_completesep = sum(do.call(rbind, lapply(sim_out,
                                  function(x) x$completesep)))/nsim
      percent_fail_MCP = sum(do.call(rbind, lapply(sim_out,
                                  function(x) x$fail_MCP)))/nsim
      percent_no_respon = sum(do.call(rbind, lapply(sim_out,
                                  function(x) x$no_respon)))/nsim
      print(c(percent_completesep, percent_fail_MCP, percent_no_respon))

      p_vals_list <- lapply(sim_out, function(x) x$p_vals)
      run_time_list <- lapply(sim_out, function(x) x$run_time)

      # Convert the list into a matrix
      sim_matrix_p_vals <- as.data.frame(t(do.call(rbind, p_vals_list)))
      run_times <- as.data.frame(t(do.call(rbind, run_time_list)))

      row.names(filtered_params) <- NULL
      # Convert the matrix to a data frame
      results <- cbind(filtered_params, RunTime = rowMeans(run_times),
                    TypeIerror_Power=apply(sim_matrix_p_vals, 1, proportion_less_than_01))
      print(results)

      filename <- paste("simulation", paramscom, "RESULTS", sep="_")
```

```
      saveRDS(results, file= filename)

      #plot
      simPlots(results, paramscom, sim_matrix_p_vals=sim_matrix_p_vals)
}

###### Power Plot Figure 2 in Paper ######
## Other Plots can be created by changing imported file names

pK_values <- as.integer(seq(0.2, 0.9, 0.1)*100)
#pK_values <- sort(as.integer(c(0.61, seq(0.2, 0.7, 0.1))*100))
#pK_values <- sort(as.integer(c(0.364,seq(0.2, 0.45, 0.05))*100))

# Initialize a list to store the last columns of the files
last_columns <- list()

# Loop through each pK value and read the corresponding RDS file
for (pK in pK_values) {
  # Construct the filename
  filename <- sprintf("/home/pinlu1/Documents/simulation_nsim_10000_nrand_1000_pK_
                    %d_rand_method_PBR_N_49_TimeTrend_FALSE_RESULTS", pK)

  # Print the filename (optional, for debugging)
  cat("Reading file:", filename, "\n")

  # Read the RDS file
  data <- readRDS(filename)

  # Extract the last column and save it in the list
  last_columns[[as.character(pK)]] <- data[[ncol(data)]]
}

# Optionally, combine all the last columns into a single data frame
# You can name the columns using the corresponding pK values
combined_last_columns <- do.call(cbind, last_columns)
colnames(combined_last_columns) <- names(last_columns)

# Specify the output file and open a PNG graphics device
pdf(file = "power_nsim_10000_nrand_1000_rand_method_PBR_N_49_TimeTrend_FALSE
                        _RESULTS.pdf", width = 10, height = 7)

# Plot the data
##colors <- c("#d7191c", "#fdae61", "#ffffbf", "#abdda4", "#2b83ba")
colors <- c(rgb(215,25,28, maxColorValue = 255),
            rgb(253, 174, 97, maxColorValue = 255),
            rgb(255, 255, 51, maxColorValue = 255),
            rgb(171, 221, 164, maxColorValue = 255),
            rgb(43, 131, 186, maxColorValue = 255))
methods <- c(
  "Population-based Test",
  "Randomisation Test (MLE)",
```

```
  "Randomisation Test (MLE; residual-based)",
  "Randomisation Test (penalized MLE)",
  "Randomisation Test (pen. MLE; residual-based)"
)
lty <- c(3,1,1,2,2)
matplot(pK_values, t(combined_last_columns), type = 'l', lty = lty, col = colors,
        lwd = 3, xlab = "Success Rate on Arm K-1 (%)", ylab = "Power",
        main = "Power Comparison of Different Tests",
        cex.main = 1.5, cex.axis = 1.2, cex.lab = 1.4)
abline(h=seq(0,1,by=0.05), lty=3, col="lightgrey")
abline(v=seq(20,90,by=10), lty=3, col="lightgrey")
matlines(pK_values, t(combined_last_columns), type = 'l', lty = lty,
                    col = colors, lwd = 2,)

# Add dots to indicate actual data points
for (i in 1:nrow(combined_last_columns)) {
  points(pK_values, combined_last_columns[i, ], col = colors[i], pch = 16, cex = 1)
}

abline(h = 0.8, col = "black", lty = 2)

# Add legend in the upper-left corner with more space between elements
legend("bottomright", legend = methods, col = colors, lty = lty, cex = 0.8,
            bty = "n", inset = 0.04, y.intersp = 1.5, lwd=2)

dev.off()

print(round(combined_last_columns*100,2))


#### Print percentage of complete separation and faild MCP-Mod
# for original data scenario but other can analysed by changing file name
filename <- sprintf("/home/pinlu1/Documents/simulation_nsim_10000_nrand_1000_pK_
            %d_rand_method_PBR_N_49_TimeTrend_FALSE_FULL", 20)

# Print the filename (optional, for debugging)
cat("Reading file:", filename, "\n")

# Read the RDS file
data <- readRDS(filename)

percent_completesep = sum(do.call(rbind, lapply(data,
                        function(x) x$completesep)))/10000
percent_fail_MCP = sum(do.call(rbind, lapply(data, function(x) x$fail_MCP)))/10000
c(percent_completesep, percent_fail_MCP)
```

runsimPMX_example.R

```
## Generate potential outcomes based on  https://doi.org/10.1002/sim.8913
## Use scenario of strong drug effect,
```

```
# 50 patients in total (Scenario III) and Emax model
## Code by Lukas Pin and Bjoern Bornkamp

# Load necessary libraries
library(DoseFinding)

source("MCP_Rand.R") ## load auxiliary code

export <- list(logit=logit, inv_logit=inv_logit, PBR = PBR,
               generate_data=generate_data, reg_mod=reg_mod, MCP_RD = MCP_RD,
               get_randomised_data=get_randomised_data,
               proportion_less_than_005=proportion_less_than_005,
               MCP_Rand=MCP_Rand, MCTtest=MCTtest,
               detect_separation=detect_separation, richtmyer=richtmyer,
               gen_pot_outcomes=gen_pot_outcomes)

gen_pot_outcomes <- function(u){
  N <- nrow(u)
  ## generate potential outcomes
  doses <- c(0, 100, 200, 400, 1000)
  ## time-point of interest
  t_end <- 12*30.4375
  ## generate individual parameters (based on quantile function)
  VA0i <- 55*exp(qnorm(u[,1], 0, sqrt(0.07)))
  VAssi <- 30*exp(qnorm(u[,2], 0, sqrt(0.2)))
  k <- 0.005*exp(qnorm(u[,3], 0, sqrt(0.2)))
  Emaxi <- 30 + qnorm(u[,4], 0, sqrt(100))
  ED50 <- 150
  eps <- qnorm(u[,5], 0, 5.3)
  Y <- matrix(nrow = N, ncol = 5)
  for(i in 1:N){
    dr <- VA0i[i] - VAssi[i]*(1 - exp(-k[i]*t_end)) + Emaxi[i]*doses/(ED50 + doses)
    Y[i,] <- dr + eps[i]
  }
  out <- data.frame(Y, base = VA0i)
  names(out)[1:5] <- paste0("Y", doses)
  out
}

#### Permuted Block Randomization ####################################
# This method is rerandomizing a sequence that was created PBR
### x is the full randomization sequence that we which to randomize
### B is the block size
PBR <- function(x, B=7){
  num_blocks <- round(length(x)/B)
  full_blocks <- num_blocks == round(length(x)/B)
  for(i in 1:num_blocks){
    x[((i-1)*B+1):(i*B)] <- sample(x[((i-1)*B+1):(i*B)])
  }
  if(!full_blocks){
    x[num_blocks*B+1:length(x)] <- sample(x[num_blocks*B+1:length(x)])
```

```
  }
  return(x)
}

# Define the custom function
proportion_less_than_005 <- function(x) {
  # Remove NAs from the input vector
  x <- na.omit(x)
  # Handle case where no data points left after omitting NAs
  if (length(x) == 0) return(NA)
  sum(x < 0.05) / length(x)
}


## generate quasi-random sequence using the Richtmyer sequence
richtmyer <- function(N, s){
  primes <- c(2,3,5,7,11,13,17,19)
  if(s > length(primes))
    stop("s too large")
  gen_vec <- sqrt(primes[1:s])
  out_mat <- matrix(nrow = N, ncol = s)
  for(i in 1:N){
    out_mat[i,] <-  (i*gen_vec)%%1
  }
  out_mat
}

# Generate quasi-random sequence
n_group <- 10
u <- richtmyer(5*n_group, 5)
plot(u[,1],u[,2])
dat <- gen_pot_outcomes(u)

# Dose levels
doses <- c(0, 100, 200, 400, 1000)

## plot individual dose-response curves
matplot(doses, t(dat[,1:5]), type="l", col = 1, lty=1, ylab="VA change")
## add true population dose-response
lines(doses, colMeans(dat[,1:5]), lwd = 3, col=2)

## generate a simulated data-set, by randomly selecting from the potential outcomes
Z <- sample(rep(1:5, each=n_group)) ## random allocation rule
resp <- dose <- numeric(5*n_group)
for(i in 1:(5*n_group)){
  dose[i] <- doses[Z[i]]
  resp[i] <- dat[i,Z[i]]
}
obs_dat <- data.frame(dose = dose, resp = resp, base = dat$base)
tapply(obs_dat$resp, obs_dat$dose, mean)
summary(lm(resp ~base, obs_dat))
```

```

# Define models for MCTtest
mods <- Mods(linear = NULL, emax = 150, sigEmax = c(175, 2),
             linlog = NULL,
             doses = doses, addArgs = list(off = 1))

# MCT Test without covariate
mm <- MCTtest(dose, resp, data=obs_dat, models = mods)
min(attr(mm$tStat, "pVal"))
#  MCT Test with adjustment for baseline
mm2 <- MCTtest(dose, resp, data=obs_dat, models = mods, addCovars = ~base)
min(attr(mm2$tStat, "pVal"))


## Scenarios
## (i) Orignial Scenario
## (ii) Order the potential outcome data by "base" (i.e. time trend in recruitment)
## (iii) Null hypothesis: Assume all potential outcomes are equal to Y(0)

## Methods to compare
## - Population MCPMod as above (with and without covariates)
## - Randomization MCP-Mod with and without covariates
#(both can be based on residual based approach)

# Main function to perform pharmacological simulations
pharmaco <- function(seed=1, rand_prod = "RA", nrand =1000){
  #Parameters
  set.seed(seed=seed)
  n_group <- 10
  u <- richtmyer(5*n_group, 5)
  dat <- gen_pot_outcomes(u)
  doses <- c(0, 100, 200, 400, 1000)
  mods <- Mods(linear = NULL, emax = 150, sigEmax = c(175, 2),
               linlog = NULL,
               doses = doses, addArgs = list(off = 1))

  # Select randomization procedure
  if(rand_prod == "RA"){
    Z <- sample(rep(1:5, each=n_group)) ## random allocation rule
  } else if (rand_prod == "PBR") {
    Z <- PBR(x=rep(c(1,1,2,2,3,3,4,4,5,5),5), B=10)   # Permuted Block Design
  }


  pvalues_all <- rep(NA,12)

  # Scenario (i) Original Scenario
  resp <- dose <- numeric(5*n_group)
  for(i in 1:(5*n_group)){
    dose[i] <- doses[Z[i]]
    resp[i] <- dat[i,Z[i]]
```

```
}

obs_dat <- data.frame(dose = dose, resp = resp, base = dat$base)

#Population MCPMod without covariates
mm <- MCTtest(dose, resp, data=obs_dat, models = mods)
pvalues_all[1] = min(attr(mm$tStat, "pVal"))

#Population MCPMod with covariates
mm2 <- MCTtest(dose, resp, data=obs_dat, models = mods, addCovars = ~base)
pvalues_all[2] = min(attr(mm2$tStat, "pVal"))

#Randomization MCP-Mod without covariates
results <- rep(0, nrand)
obvs <- MCTtest(dose, resp, data=obs_dat, models = mods)$tStat
for(j in 1:nrand){
  datR <- get_randomised_data(obs_dat[, 1:2], randMethod= rand_prod, doses, B=10,
                              N=50, n=rep(10,5))
  results[j] <- max(MCTtest(dose, resp, data=datR, models = mods)$tStat)
}
pvals <- rep(0,4)
for(i in 1:4){
  pvals[i]<- sum(results- obvs[i]>= 0 )/nrand
}
pvalues_all[3] = min(pvals)

#Randomization MCP-Mod with covariates
results <- rep(0, nrand)
obvs <- MCTtest(dose, resp, data=obs_dat, models = mods, addCovars = ~base)$tStat
for(j in 1:nrand){
  datR <- get_randomised_data(obs_dat[, 1:3], randMethod=rand_prod, doses, B=10,
                              N=50, n=rep(10,5))
  results[j] <- max(MCTtest(dose, resp, data=datR, models = mods,
                              addCovars = ~base)$tStat)
}
pvals <- rep(0,4)
for(i in 1:4){
  pvals[i]<- sum(results- obvs[i]>= 0 )/nrand
}
pvalues_all[4] = min(pvals)

# Scenario (ii) Order the potential outcome data by "base"
dat_T <- dat[order(dat$base), ]
resp <- dose <- numeric(5*n_group)
for(i in 1:(5*n_group)){
  dose[i] <- doses[Z[i]]
  resp[i] <- dat_T[i,Z[i]]
}
obs_dat <- data.frame(dose = dose, resp = resp, base = dat_T$base)

#Population MCPMod without covariates
```

```
mm <- MCTtest(dose, resp, data=obs_dat, models = mods)
pvalues_all[5] = min(attr(mm$tStat, "pVal"))

#Population MCPMod with covariates
mm2 <- MCTtest(dose, resp, data=obs_dat, models = mods, addCovars = ~base)
pvalues_all[6] = min(attr(mm2$tStat, "pVal"))

#Randomization MCP-Mod without covariates
results <- rep(0, nrand)
obvs <- MCTtest(dose, resp, data=obs_dat, models = mods)$tStat
for(j in 1:nrand){
  datR <- get_randomised_data(obs_dat[, 1:2], randMethod=rand_prod, doses, B=10,
                              N=50, n=rep(10,5))
  results[j] <- max(MCTtest(dose, resp, data=datR, models = mods)$tStat)
}
pvals <- rep(0,4)
for(i in 1:4){
  pvals[i]<- sum(results- obvs[i]>= 0 )/nrand
}
pvalues_all[7] = min(pvals)

#Randomization MCP-Mod with covariates
results <- rep(0, nrand)
obvs <- MCTtest(dose, resp, data=obs_dat, models = mods, addCovars = ~base)$tStat
for(j in 1:nrand){
  datR <- get_randomised_data(obs_dat[, 1:3], randMethod=rand_prod, doses, B=10,
                              N=50, n=rep(10,5))
  results[j] <- max(MCTtest(dose, resp, data=datR, models = mods,
                      addCovars = ~base)$tStat)
}
pvals <- rep(0,4)
for(i in 1:4){
  pvals[i]<- sum(results- obvs[i]>= 0 )/nrand
}
pvalues_all[8] = min(pvals)

## (iii) Null hypothesis: Assume all potential outcomes are equal to Y(0)
dat_N <- dat
dat_N[,2:5] <- dat_N[,1]
resp <- dose <- numeric(5*n_group)
for(i in 1:(5*n_group)){
  dose[i] <- doses[Z[i]]
  resp[i] <- dat_N[i,Z[i]]
}
obs_dat <- data.frame(dose = dose, resp = resp, base = dat_N$base)

#Population MCPMod without covariates
mm <- MCTtest(dose, resp, data=obs_dat, models = mods)
pvalues_all[9] = min(attr(mm$tStat, "pVal"))

#Population MCPMod with covariates
```

```
  mm2 <- MCTtest(dose, resp, data=obs_dat, models = mods, addCovars = ~base)
  pvalues_all[10] = min(attr(mm2$tStat, "pVal"))
  results <- rep(0, nrand)

  #Randomization MCP-Mod without covariates
  obvs <- MCTtest(dose, resp, data=obs_dat, models = mods)$tStat
  for(j in 1:nrand){
    datR <- get_randomised_data(obs_dat[, 1:2], randMethod=rand_prod, doses, B=10,
                            N=50, n=rep(10,5))
    results[j] <- max(MCTtest(dose, resp, data=datR, models = mods)$tStat)
  }
  pvals <- rep(0,4)
  for(i in 1:4){
    pvals[i]<- sum(results- obvs[i]>= 0 )/nrand
  }
  pvalues_all[11] = min(pvals)

  #Randomization MCP-Mod with covariates
  results <- rep(0, nrand)
  obvs <- MCTtest(dose, resp, data=obs_dat, models = mods, addCovars = ~base)$tStat
  for(j in 1:nrand){
    datR <- get_randomised_data(obs_dat[, 1:3], randMethod=rand_prod, doses, B=10,
                                N=50, n=rep(10,5))
    results[j] <- max(MCTtest(dose, resp, data=datR, models = mods,
                          addCovars = ~base)$tStat)
  }
  pvals <- rep(0,4)
  for(i in 1:4){
    pvals[i]<- sum(results- obvs[i]>= 0 )/nrand
  }
  pvalues_all[12] = min(pvals)
  return(pvalues_all)
}

grid <- expand.grid(
  rand_prod = c("RA", "PBR") ,
  KEEP.OUT.ATTRS = FALSE,
  stringsAsFactors = FALSE)


for (i in 1:nrow(grid)) {
  param_constatnt <- list(nrand=1000, rand_prod= grid[i,])

  nsim = 10000
  seeds <- data.frame(seed=seq(1,nsim))

  sim_out <- Q_rows(fun = pharmaco,
                  df = seeds,
                  const = param_constatnt,
                  seed = 23456,
                  n_jobs = nrow(seeds),
```

```
                          template = list(
                            walltime = 300,
                            job_name = "Pharmaco",
                            log_file = "logs.txt"),
                          pkgs = c("data.table", "DoseFinding", "logistf"),
                          export = export)

  filename <- paste("simulation_pharmaco", grid[i,], param_constatnt$nrand, "FULL",
                              sep="_")
  saveRDS(sim_out, file= filename)

  sim_matrix_p_vals <- as.data.frame(t(do.call(rbind, sim_out)))

  results <- cbind(TypeIerror_Power=apply(sim_matrix_p_vals, 1,
                              proportion_less_than_005))
  print(results)

  filename <- paste("simulation_pharmaco", grid[i,], param_constatnt$nrand,
                              "RESULTS", sep="_")
  saveRDS(results, file= filename)
}
```
