# Supplementary figures and images for: Randomization‐Based Inference for MCP‐Mod
Source: Stat Med. 2025 May 22;44(10-12):e70092. doi: 10.1002/sim.70092 (PMC12097294; doi:10.1002/sim.70092)

Model means

emax (ED50=10)

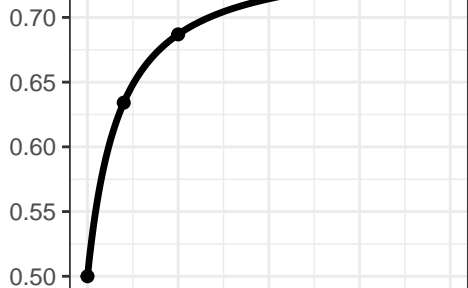

emax (ED50=50)

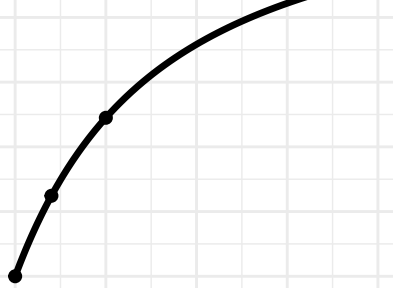

sigEmax (ED50=5,h=3)

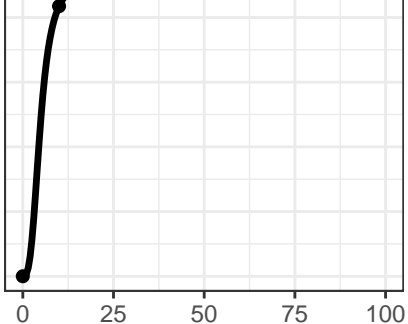

sigEmax (ED50=25,h=3)

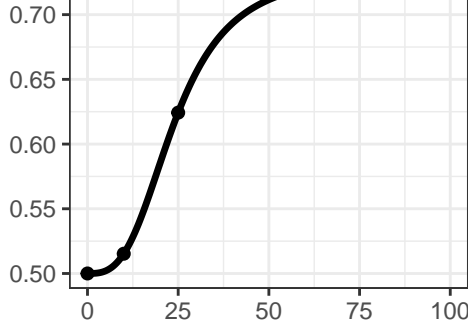

betaMod  
delta1=0.2475,delta2=2.025,scal=200

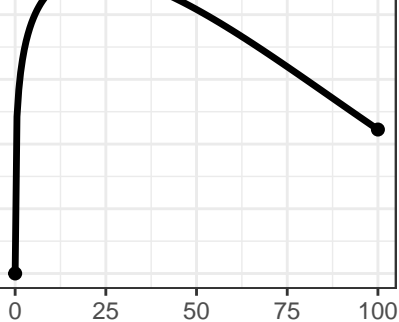

Dose

Supplement: Supplementary file 2 — Data. Supporting Information. [file SIM-44-0-s001.zip › simulations/Models.pdf]
